# Systematic review: Perceptions of type 2 diabetes of people of African descent living in high‐income countries

**DOI:** 10.1111/jan.15266

**Published:** 2022-04-20

**Authors:** Onuorah Love, Draper Peter, Santy‐Tomlinson Julie

**Affiliations:** ^1^ Staffordshire University Stoke‐on‐Trent UK; ^2^ University of Hull Hull UK; ^3^ Odense University Hospitals Odense Denmark; ^4^ University of Southern Denmark Odense Denmark

**Keywords:** African Americans, Africans, attitudes, behaviours, beliefs, Caribbean, diabetes, nursing, perceptions, systematic literature review

## Abstract

**Aims:**

To describe how people of African descent perceive and understand type 2 diabetes, and to examine the impact of their perceptions and beliefs on the uptake of diet, exercise, weight control and adherence to medication recommendations.

**Design:**

Systematic literature review of quantitative and qualitative studies.

**Data sources:**

We searched MEDLINE, CINAHL Complete, Psych INFO, Academic Search Premier, Education Research Complete, Web of Science and Scopus, World Health Organization (WHO), Diabetes UK and American Diabetes Association for articles published from January 1999 to December 2019.

**Review methods:**

Informed by the PRISMA guidelines, we independently reviewed titles and abstracts, identified articles for full‐text review that met inclusion criteria, conducted a quality assessment and extracted data. Findings were synthesized using a thematic approach.

**Results:**

Twenty‐six studies met the inclusion criteria. Knowledge and understanding of diabetes were poor. Beliefs and behaviours about diet, exercise, weight and health care were erroneous. Most diabetic participants could not recognize diabetes symptoms, failed to take their diagnosis seriously and did not adhere to medication recommendations. The resultant effect was an increased risk of complications with undesirable outcomes.

**Conclusion:**

Poor diabetes perceptions are linked to negative consequences and may be responsible for poorer outcomes among people of African descent. This review highlights the need to consider this population's beliefs and practices in structuring culturally sensitive programmes for diabetes management.

**Impact:**

This systematic literature review is the first to exclusively explore perceptions of people of African descent in relation to diabetes. It is important to consider people of African descents' diabetes perceptions and practices before formulating interventions for their diabetes management.

## 1 INTRODUCTION

The incidence of diabetes continues to rise worldwide, especially among people of African descent. Despite various educational programmes to enhance prevention and self‐management, people of African descent suffer worse outcomes than other ethnicities (Assari et al., 2017). Perceptions of health and illness influence behaviour and attitudes towards conditions like diabetes (Diefenbach & Leventhal, 1996; Langston et al., 2017). Accurate perceptions of diabetes increase individual adherence to educational programmes (Attridge et al., 2014; Seear et al., 2019). Hence, a clear understanding of how health and illness are individually or collectively perceived may enhance development of interventions to improve diabetes self‐care and overall quality of life for this population.

## 2 BACKGROUND

Diabetes is a chronic, metabolic disorder characterized by excessive accumulation of blood glucose leading to serious damage to the heart, blood vessels, eyes, kidneys and nerves over time. Type 2 diabetes is the most common, usually occurring in adults when the body either does not respond to, or does not make enough insulin (WHO, 2021). Reasons for developing diabetes are multifactorial, including genetic susceptibility, metabolic disorder and environmental factors such as diet, physical activity and obesity (Golden et al., 2019). People of African descent (sub‐Saharan African, African Caribbean and African American) living in high‐income countries, irrespective of their migration periods or history, show high incidence of this condition and suffer worse outcomes (Assari et al., 2017). Lifestyle changes can prevent and/or delay the onset of type 2 diabetes (Glechner et al., 2018; Kriska et al., 2018; Siddiqui et al., 2018; Uusitupa et al., 2019). However, willingness and readiness to engage in necessary lifestyle changes depend on an individual's or group's perspectives about diabetes. Individual's beliefs and perceptions are influenced by their cultural and social backgrounds and experiences which, in turn, influence self‐management behaviour (Jakub et al., 2018; Shiyanbola et al., 2018). Policies based on studies exploring perceptions have been shown to be more effective in empowering high‐risk populations to adopt relevant diabetes management life changes (Attridge et al., 2014; Seear et al., 2019).

Exploring perceptions of diabetes, causes and effective management interventions of people of African descent, therefore, is fundamental to develop effective preventive and self‐management interventions required for a chronic condition like diabetes.

Nurses, the largest group of healthcare professionals worldwide, play varied roles in health promotion, illness prevention, needs assessment, planning, implementing and evaluating interventions to improve the safety and quality of care in diverse settings. Hence, they are likely to be the first group of providers to come in contact with individuals at risk of or diagnosed with diabetes. Awareness of perceptions and beliefs about diabetes among people of African descent may facilitate high‐quality care and improved health outcomes for this population.

We found no published literature summarizing the current state of knowledge in this area. Our overall purpose, therefore, was to summarize perceptions and understanding of type 2 diabetes among people of African descent to inform efforts to develop healthcare interventions that will improve outcomes in this population.

## 3 AIMS

Specific study aims were:

1. To describe how people of African descent perceive and understand type 2 diabetes; and
2. To examine the impact of their perceptions and beliefs on the uptake of diet, exercise, weight control and adherence to medication recommendations.

## 4 REVIEW QUESTION

Our specific research questions were:

1. What are the perceptions and beliefs of people of African descent in relation to diabetes, causes and type 2 diabetes effective management interventions?
2. What impact do their perceptions and beliefs about diabetes have on the uptake of diet, exercise, weight‐management and use of healthcare?

## 5 METHODS

### 5.1 Search strategy

**FIGURE 1:**
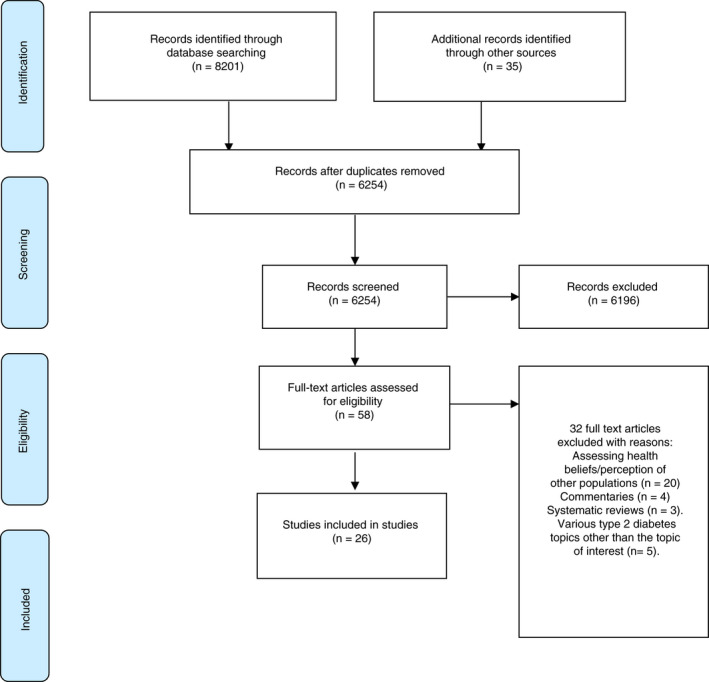
The artwork demonstrates the process of the study selection guided by the preferred reporting items for systematic reviews and meta‐analysis (PRISMA) statement guideline (Moher et al., 2015)

This review was informed by the Cochrane Handbook version 6.1 (Higgins et al., 2019). The search strategy was developed using the Preferred Reporting Items for Systematic Reviews and Meta‐Analysis (PRISMA) guidelines (Moher et al., 2015). Due to the scarcity of literature on this topic, all original research studies published in the English language that included people of African descent over 18 years of age, with or without type 2 diabetes, were included. Systematic, scoping and literature reviews, commentaries, opinions, editorials and studies focused on diabetes other than type 2 were excluded (Figure 1).

### 5.2 Search methods

We searched MEDLINE, CINAHL Complete, Psych INFO, Academic Search Premier, Education Research Complete, Web of Science and Scopus, World Health Organization (WHO), Diabetes UK and American Diabetes Association for English Language articles published between January 1999 and December 2019. Reference lists of retrieved articles were hand searched for additional publications. Boolean operators were used as links to identify relevant keywords and phrases. Truncation symbols (*) were used to identify variations in root words (diabet*; percept*). Wild cards (?) and (#) were used to capture words with unknown characters and those with different spellings respectively as applicable for each database and website.

Mixed‐method systematic reviews preserve the originality of the findings (Hemingway & Brereton, 2009) without loss of scientific rigour (Harden & Thomas, 2005). Therefore, due to the scarcity of literature, we included all qualitative and quantitative designs to provide a broad perspective on the topic.

Studies initially underwent title and abstract screening, then a full‐text review. Results were independently cross‐checked by the three authors. The authors discussed and resolved any differences concerning inclusion or exclusion of studies. Further screening of the studies was conducted by three other independent reviewers and one more article was retrieved. The search strategy, inclusion and exclusion criteria, and data extraction elements are presented in Appendix S1.

### 5.3 Quality appraisal

Methodological quality of the included articles was assessed by LO and independently reviewed by PD and JST. Two quality assessment tools, the Critical Review Form version 2 for qualitative studies (Letts et al., 2007a; Letts et al., 2007b) and the Guidelines for Critical Review Forms for quantitative Studies (Law et al., 1998) were adapted for this review. Both tools are well‐defined with clear instructions on how each question should be answered. We merged the components of both tools which were the same except for ‘outcome’, ‘intervention’ and ‘results’ applicable only to the quantitative studies.

Sets of questions for each component of the quality assessment tools were rated: (1) ‘very likely’; (2) ‘somewhat likely’; (3) ‘not likely’; and (4) ‘can't tell’. For example, critical questions to determine the sampling strength were: ‘whether the study described the purposive sampling process’; ‘whether sampling continued until saturation’; and ‘whether informed consent was obtained’. Where each of these questions within ‘sampling strength’ was scored 1, sampling for that article was scored ‘strong’. The overall quality of each study was determined by the number of ‘strong’ ratings assigned. Four ‘strong’ ratings without a ‘weak’ rating were scored ‘strong’. Moderate scores were assigned to studies with less than four strong ratings plus 1 weak rating. Two or more weak ratings in a study indicated a weak score. However, data from all the studies were included.

### 5.4 Data extraction

The data extraction form was derived from the aims of this review (see Appendix S1). The appropriateness of the extraction form was tested on two of the studies prior to use (Higgins et al., 2011), and was satisfactory.

### 5.5 Data synthesis

An inductive thematic approach to data synthesis was used (Braun & Clarke, 2006). Each of the studies was read and key words of the findings were recorded on a spreadsheet by LO. Words with similar meanings were grouped to form codes. Codes were then organized into themes and subthemes. The process was independently cross‐checked by PD and JST.

## 6 FINDINGS

### 6.1 Methodological descriptions

The search initially retrieved 8236 articles. After removing duplicates, titles and abstracts of 6254 articles were screened for eligibility after which 58 articles remained. After full‐text review, 26 studies met inclusion criteria. Of the included studies, 17 were qualitative (Gore, 1999; Liburd et al., 1999; Skelly et al., 2006; Wenzel et al., 2006; Brown et al., 2007; Peek et al., 2010; Wallin & Ahlström, 2010; Yeoh & Furler, 2011; Breland et al., 2013; Balls‐Berry et al., 2015; Bockwoldt et al., 2016; Cooper & Lemonde, 2016; Issaka et al., 2016; Okop et al., 2016; Moise et al., 2017; Babatunde‐Sowole et al., 2018; Cameron et al., 2018), eight were cross‐sectional survey/questionnaire (Wallhagen & Lacson, 1999; Stover et al., 2001; Ford et al., 2002; Mann et al., 2009; Abubakari et al., 2011; Calvin et al., 2011; Hyman et al., 2014; Foster et al., 2016), and one cross‐sectional randomized clinical trial (Baptiste‐Roberts et al., 2006) (Table 1). No study comprising of mixed methodology was retrieved. Six of these studies were of ‘moderate’ quality, one scored ‘weak’ and the rest were identified as ‘strong’.

**TABLE 1:** Summary of included articles

Authors/year	Aim of study	Design/method	Sample	Ethnicity	Country of study
Abubakari et al. 2011	To investigate the relationship between diabetes‐specific knowledge, illness perceptions and self‐management behaviours and the relationship between self‐management behaviours and metabolic control	Cross‐sectional questionnaire	*N* = 359 (genders not specified)	Black Caribbean, Black Africans & White British	UK
Babatunde‐Sowole et al., 2018	To explore the diet and lifestyle changes contributing to weight gain among Australian West African women following migration	Qualitative storytelling approach	*N* = 20 (female, *n* = 20)	West African immigrant women	Australia
Balls‐Berry et al., 2015	To assess knowledge, attitude and behaviour about diabetes prevention programme and development	Focus group	*N* = 13 (male, *n* = 13)	African American	US
Baptiste‐Roberts et al., 2006	To assess perception of body image	Cross‐sectional RCT	*N* = 185 (female, *n* = 141), (male, *n* = 44)	African American	US
Bockwoldt et al., 2016	To describe the lived experiences of African Americans adapting to prescribed medications for type 2 diabetes and to identify factors that influenced adaptation	Focus group	*N* = 13 (female, *n* = 10), (male, *n* = 3)	African American	US
Breland et al., 2013	To assess effects of race and ethnicity on eating and to explore food types, preparation, sources and meal communication with providers	Grounded theory	*N* = 37 (female, *n* = 27), (male, *n* = 10)	African American & Latinos	US
Brown et al., 2007	To understand the impact of health beliefs, diabetes associated risks and expectation of treatments on diabetes management	Individualized interview	*N* = 16 (female, *n* = 10), (male, *n* = 6)	Caribbean	UK
Calvin et al., 2011	To describe perceived risk for diabetes complications	Cross‐sectional survey/questionnaire	*N* = 143 (female, *n* = 76), male, *n* = 67)	African American	US
Cameron et al., 2018	To understand perception of Health, Body image and attractiveness	Individualized semi structured interview	*N* = 11 (female, *n* = 11)	African American	US
Cooper and Lemonde, 2016	To explore health beliefs held by adult African immigrants about diabetes and their practices in preventing it	Focus group's semi structured interview	*N* = 14 (female, *n* = 11), (male, *n* = 3)	West African immigrants	Canada
Ford et al., 2002	To assess perception of diabetes of African Americans and White Americans and to examine psychometric properties of instruments used to measure perception	Questionnaire	*N* = 45 (gender not specified)	African American & Whites	US
Foster et al., 2016	To assess knowledge, beliefs and practices, health‐seeking behaviours and self‐management and attitude of patients with diabetic retinopathy	Cross‐sectional questionnaire	*N* = 150 (female, *n* = 99), (male, *n* = 51)	West Indies	West Indies
Gore et al. 1999	To explore perception of ideal body weight	Focus group	*N* = 55 (female, *n* = 55)	African American	US
Hyman et al., 2014	To explore self‐management practices, use of diabetes information and heath service for diabetes care	Cross‐sectional questionnaire	*N* = 102 (gender not specified)	Caribbean immigrants & Canadian born	Canada
Issaka et al., 2016	To explore perception of type 2 diabetes	Focus group	*N* = 61 (gender not specified)	African immigrants	Australia
Liburd et al., 1999	To explore perception of body size and shape of black women	Focus group	*N* = 33 (female, *n* = 33)	African American	US
Mann et al., 2009	To identify suboptimal diabetes knowledge and beliefs, medication and monitoring that may be hindrances to effective self‐management	Cross‐sectional survey/questionnaire	N = 151 (gender not specified)	African American & Latinos	US
Moise et al., 2017	To generate culturally informed insight into diabetes knowledge, management and prevention	Focus group	*N* = 10 (female, *n* = 7), (male, *n* = 3)	Haitian immigrants	US
Okop et al., 2016	To explore perception of body size, obesity risk and willingness to lose weight	Focus group	*N* = 78 (female, *n* = 36), (male, *n* = 42)	Black South African	South Africa
Peek et al., 2010	To explore perception of the influence of race on shared decision‐making (SDM)	Phenomenological approach	*N* = 51 (female, *n* = 42), (male, *n* = 9)	African American	US
Skelly et al., 2006	To understand the views and prevention of diabetes	Ethnographic interview	*N* = 42 (female, *n* = 22), (male, *n* = 20)	African American	US
Stover et al., 2001	To explore health perception and their relationship to symptoms	Questionnaire	*N* = 75 (gender not specified)	African American	US
Wallhagen and Lacson, 1999	To explore perception of sense of control of type 2 diabetes	Cross‐sectional questionnaire	*N* = 23 (female, *n* = 11), male, *n* = 12)	African American	US
Wallin and Ahlström, 2010	To investigate experience of diabetes diagnosis and to investigate how they describe their health beliefs	Individualized interview	*N* = 19 (female, *n* = 11), (male, *n* = 8)	Somalis	Sweden
Wenzel et al., 2006	To explore the experience of being diagnosed with diabetes	Focus group	*N* = 73 (female, *n* = 42), (male, *n* = 31)	African American	US
Yeoh and Furler, 2011	To explore diabetes perception within the context of broader health circumstances	Grounded theory	*N* = 25 (female, *n* = 19), (male, *n* = 6)	Sudanese	Australia

Sixteen of the studies were conducted in the US, three in Australia, two each in Canada and the UK and there was one study each from Sweden, South Africa and the West Indies. The sample size ranged from 10 to 359 with a total of 1804 participants comprising both diabetics and non‐diabetics. Some articles only included immigrants while some only had host‐country citizens. Because most of the studies were undertaken in the US, most of the participants were African Americans. Tables 1 and 2 summarize the included studies, themes and subthemes of the review. Three themes: 1. Diabetes perceptions and knowledge, 2. Consequences of poor diabetes perceptions and knowledge, 3. Beliefs about the causes of diabetes and 12 subthemes were identified from the review.

**TABLE 2:** Themes & subthemes

Themes	Subthemes	Articles
Diabetes Perceptions & Knowledge	Diabetes knowledge	Moise et al., 2017; Cooper & Lemonde, 2016; Issaka et al., 2016; Abubakari et al., 2011; Calvin et al., 2011; Yeoh & Furler, 2011; Mann et al., 2009; Skelly et al., 2006; Wenzel et al., 2006; Ford et al., 2002
	Weight perception & diabetes	Babatunde‐Sowole et al., 2018; Cameron et al., 2018; Issaka et al., 2016; Okop et al., 2016; Baptiste‐Roberts et al., 2006; Skelly et al., 2006; Liburd et al., 1999; Gore et al., 1999
	Exercise perception & diabetes	Skelly et al., 2006; Cooper & Lemonde, 2016; Foster et al., 2016
	The Role of diet	Babatunde‐Sowole et al., 2018; Cooper & Lemonde, 2016; Foster et al., 2016; Breland et al., 2013; Mann et al., 2009; Brown et al., 2007; Skelly et al., 2006
	Risk perception & Symptom recognition	Foster et al., 2016; Balls‐Berry et al., 2015; Calvin et al., 2011; Mann et al., 2009; Brown et al., 2007; Skelly et al., 2006; Wenzel et al., 2006; Stover et al., 2001; Wallhagen & Lacson, 1999
	Diagnosis	Wallin & Ahlstrom, 2010; Wenzel et al., 2006
	Medication perception	Bockwoldt et al., 2016; Brown et al., 2007; Mann et al., 2009; Skelly et al., 2006; Wenzel et al., 2006
	Perception of spiritual influence	Copper and Lemonde, 2016; Issaka et al., 2016; Wallin & Ahlstrom, 2010; Mann et al., 2009; Brown et al., 2007; Skelly et al., 2006; Wenzel et al., 2006
Consequences of Poor Perceptions & Knowledge of Diabetes	Increased diabetes symptoms and risk of complications	Liburd et al., 1999; Wallhagen & Lacson, 1999; Stover et al., 2001; Ford et al., 2002; Skelly et al., 2006; Baptiste‐Roberts et al., 2006; Mann et al., 2009; Calvin et al., 2011; Hyman et al., 2014 Okop et al., 2016; Issaka et al., 2016; Foster et al., 2016
Beliefs about the Causes of Diabetes	Cultural & environmental effect	Brown et al., 2007; Yeoh & Furler, 2011; Hyman et al., 2014; Issaka et al., 2016; Moise et al., 2017
	Racial discrimination & stress	Brown et al., 2007; Yeoh & Furler, 2011; Breland et al., 2013; Issaka et al., 2016; Cooper & Lemonde, 2016
	Communication & information sharing	Yeoh & Furler, 2011; Skelly et al., 2006; Brown et al., 2007; Peek et al., 2010; Breland et al., 2013; Hyman et al., 2014; Balls‐Berry et al., 2015; Cameron et al., 2018

### 6.2 Diabetes perceptions and knowledge

#### 6.2.1 Diabetes knowledge

Overall, poor knowledge of diabetes was reported. For example, 43% of the participants in Foster et al.'s (2016) study were unable to explain diabetes. Participants demonstrated general awareness about diabetes (Issaka et al., 2016; Moise et al., 2017; Yeoh & Furler, 2011) but specific knowledge about risk factors and susceptibility to diabetes was deficient (Balls‐Berry et al., 2015; Mann et al., 2009; Skelly et al., 2006). Knowledge about the role of genetic/hereditary factors and behaviours like the consumption of unhealthy food and lack of exercise was expressed (Cooper & Lemonde, 2016; Issaka et al., 2016; Skelly et al., 2006), but some reasoned that diabetes was unavoidable because of genetic causes (Skelly et al., 2006). The effects of diabetes were mistaken for the cause of diabetes. Some believed it was curable (Mann et al., 2009) or would spontaneously disappear, thus not connecting diabetes complications with poor management (Ford et al., 2002). Half the participants in Calvin et al.'s study (Calvin et al., 2011) did not consider diabetes to be chronic. Diabetes was perceived to be present only when the blood sugar level was high, and absent when it was low (Mann et al., 2009). Most African immigrants considered diabetes less serious than HIV/AIDS and cancer and paid less attention to the disease (Issaka et al., 2016).

#### 6.2.2 Weight perception and diabetes

Perceptions of overweigh/obesity varied (Baptiste‐Roberts et al., 2006; Cameron et al., 2018; Issaka et al., 2016; Liburd et al., 1999; Okop et al., 2016; Skelly et al., 2006). Liburd et al. (1999) assessed body size and shape of black womens' body silhouettes. Participants showed preferences for small to middle‐sized silhouettes, while perceiving middle‐sized silhouettes as healthiest. Some considered smaller body size next to their current weight as ideal (Okop et al., 2016). Others felt good or bad about their current weight based on their levels of satisfaction. Some obese and optimal weight participants with low satisfaction levels viewed obesity as threat to their health (Okop et al., 2016). Many overweight and obese participants were satisfied with their body size (Baptiste‐Roberts et al., 2006; Cameron et al., 2018; Okop et al., 2016). Some argued that no image is ideal and rejected the use of BMI in measuring health or unhealthiness (Cameron et al., 2018).

Obesity was not identified by some as a factor in diabetes susceptibility (Baptiste‐Roberts et al., 2006; Issaka et al., 2016; Okop et al., 2016; Skelly et al., 2006). Some considered obesity as evidence of good health and a mark of affluence (Babatunde‐Sowole et al., 2018; Issaka et al., 2016; Okop et al., 2016). For some, obesity signified happiness and no threat to health (Okop et al., 2016) and was thought to be ideal for women (Baptiste‐Roberts et al., 2006; Liburd et al., 1999). For example, in the body image perception study by Baptiste‐Roberts et al. (2006) the desired weight for the female participants and the males' perceptions of the ideal weight for the females were BMI 27.7 and 28.3, respectively (normal range – 18.5 to 24.9).

Thinness was associated with health conditions like HIV/AIDS, cancer or psychological issues like depression (Okop et al., 2016). Some perceived diabetes as causing weight loss (Skelly et al., 2006). These views were reflected in participants' preferences for heavy‐sized bodies (Skelly et al., 2006). Both genders in Baptiste‐Robert et al.'s study (Baptiste‐Roberts et al., 2006) believed that persons of the same gender should become heavier as their ages increased.

#### 6.2.3 Exercise perception and diabetes

Lack of understanding of the link between exercise and diabetes was also apparent (Foster et al., 2016; Skelly et al., 2006), even among those who believed in the importance of exercise (Cooper & Lemonde, 2016; Foster et al., 2016). In one study, only a small fraction of participants actually exercised regularly (Foster et al., 2016).

#### 6.2.4 The role of diet

The importance of diet was discussed in some studies (Foster et al., 2016; Skelly et al., 2006), but there was limited knowledge of what a healthy diet entailed (Foster et al., 2016; Skelly et al., 2006). Understanding the effect of diet modification on blood glucose was lacking and home remedies were commonly used to make up for the limited dietary knowledge (Breland et al., 2013). Participants' ideas of healthy eating were influenced by their culture and experiences. For example, some considered dietary restrictions for diabetics harmful, arguing that the hunger and craving for sugar that is associated with diabetes should be met to avoid emotional instability (Breland et al., 2013). Eating small portions were believed to be responsible for causing other health issues including hypoglycaemic symptoms that were perceived as worse than hyperglycaemia (Breland et al., 2013; Brown et al., 2007). Participants often relied on subjective feelings of blood glucose to control their diabetes (Brown et al., 2007; Mann et al., 2009). Others deliberately maintained a high blood glucose level for convenience and many ate frequently to avoid feelings of weakness (Brown et al., 2007; Mann et al., 2009).

Dietary recommendations were abandoned when there was no immediate improvement in expected outcomes (Breland et al., 2013). Dietary advice given by healthcare workers was seen as suboptimal because they did not understand patients' needs and dietary preferences (Breland et al., 2013; Brown et al., 2007). Some participants believed that clinicians' prescriptions and dietary recommendations were generic, unfamiliar or unrealistic and, so, rejected them. The use of intuition to substitute for unclear food advice was reported; for example, participants would skip meals to substitute for eating less (Breland et al., 2013). Some argued that the diet they were asked to give up did not make them sick in the past, while others believed that their blood glucose went up regardless of what they ate (Breland et al., 2013). Participants were more willing to discuss treatment with medication than with diet change (Wenzel et al., 2006).

The impact of families on food choice was also identified. Some participants stated that they chose to eat the same unhealthy food as their families because their families were unwilling to adapt to the healthy diabetic diet. Eating different meals from the family menu was isolating and costly (Breland et al., 2013). Others expressed that the location of healthy food stores/shops made it difficult to access them due to transportation, cost and time, forcing reliance on the unhealthy foods that were readily accessible (Breland et al., 2013).

#### 6.2.5 Risk perception and symptom recognition

Risk perception and symptom recognition were shown to be deficient in most of the studies (Balls‐Berry et al., 2015; Brown et al., 2007; Calvin et al., 2011; Foster et al., 2016; Mann et al., 2009; Skelly et al., 2006). Most participants stated that it was difficult to take the condition seriously even after they had been diagnosed and they only realized the need for treatment after experiencing serious diabetes‐related illnesses (Wenzel et al., 2006). Some perceived that diabetes has few symptoms with minimal consequences, while most had not heard about HbA1c (Mann et al., 2009). Nephropathy, amputation and retinopathy/blindness were the least perceived risks to their health (Calvin et al., 2011; Foster et al., 2016). Sixty percent of participants in Foster et al.'s (2016) study were unaware of retinopathy screening at the time of diabetes diagnosis, half did not know they required annual screening and most of the male participants thought that they could only see the ophthalmologist when their vision was impaired.

#### 6.2.6 Diagnosis

Many of the diabetic participants stated that they had experienced symptoms for several years prior to being diagnosed after suffering major symptoms such as loss of consciousness (Wallin & Ahlström, 2010; Wenzel et al., 2006). Some were diagnosed through symptom manifestations such as excessive thirst and urination (Wenzel et al., 2006) which were sometimes detected after several visits to the doctor (Wallin & Ahlström, 2010). A few found out the diagnosis during routine check‐ups (Wallin & Ahlström, 2010; Wenzel et al., 2006) or through treatment for other conditions. Some individuals found out through the use of their friend's or family member's glucometer (Wenzel et al., 2006). The news of diagnosis was received with varied emotions. Most were not surprised because of diabetes prevalence among their family and friends (Wenzel et al., 2006). Others described their reaction of shock, fear, anxiety and the tendency to doubt the diagnosis where no symptoms existed (Wallin & Ahlström, 2010). The attitudes of denial and refusal to acquire diabetes knowledge were adopted to forget the diagnosis (Wallin & Ahlström, 2010). Most immigrant participants considered the positive consequences as favourable; for example, giving up sweets was considered advantageous. They also took solace from the belief that normal life could still be maintained as a diabetic in their host countries with stable economies, a wide knowledge of the disease and the availability of medication (Wallin & Ahlström, 2010; Wenzel et al., 2006). Positive comparisons of type 2 with type 1 diabetes and other conditions that were thought to be more dangerous were used as techniques to cope with the diagnosis (Wallin & Ahlström, 2010).

#### 6.2.7 Medication perception

Views about the use of diabetes medications differed. The severity of the disease was measured by the type of treatment that was recommended (Wenzel et al., 2006). Glycaemic control with diet and medication were understood as a less serious case of type 2 diabetes (Bockwoldt et al., 2016; Brown et al., 2007; Skelly et al., 2006). Insulin use was perceived as being for more serious cases (Bockwoldt et al., 2016; Brown et al., 2007), and was, therefore, surprising and harder to accept (Bockwoldt et al., 2016). Disappointment, shame, anxiety, frustration, despondency and self‐blame were attributed to insulin initiation, perceived as indicating failure to take care of self (Bockwoldt et al., 2016). Some believed that it was restrictive, caused weight gain and indicated that their condition was getting worse with imminent complications, others feared the injection (Brown et al., 2007).

Two studies (Brown et al., 2007; Mann et al., 2009) explored non‐compliance with diabetes medications. Participants were non‐compliant with treatment advice that did not conform to their health beliefs (Brown et al., 2007). Worries about side effects of diabetes medications (Brown et al., 2007; Mann et al., 2009) and possible addictions were expressed along with complaints about the medication being hard to swallow (Mann et al., 2009). Some participants believed that medication could be skipped when blood glucose levels were ‘normal’ (Mann et al., 2009). Caribbean participants believed that the traditional medicines they used in their homeland were more effective than western medicine (Brown et al., 2007). There were beliefs that medications could cure diabetes. Physical activity and weight loss were misunderstood by some as only necessary when diagnosed with diabetes, but not also for prevention (Skelly et al., 2006). Those who believed that insulin was an ideal treatment for the disease and those who feared the risk of complications accepted insulin while others did not accept that they could develop complications (Brown et al., 2007).

#### 6.2.8 Perception of spiritual influence

Perceptions of spiritual influence were linked to experiences with the diagnosis, acceptance of the condition and their trust in God's help in managing it (Brown et al., 2007; Cooper & Lemonde, 2016; Issaka et al., 2016; Skelly et al., 2006; Wallin & Ahlström, 2010; Wenzel et al., 2006). Some participants expressed a sense of powerlessness (fatalism) over their condition which they saw as the will of God and not within their control (Issaka et al., 2016; Mann et al., 2009) while most African immigrants believed it was due to unseen forces or sheer bad luck (Issaka et al., 2016). Many trusted God for the outcome of their diabetes although, for some, this understanding did not hinder them from seeking medical treatment (Brown et al., 2007; Wenzel et al., 2006). Older adults expressed the belief that God could cure their diabetes through behaviour change and by the work of the doctors (Skelly et al., 2006). Some stated that illness came from God and that asking why could imply ingratitude, lack of faith and sinfulness, and may result in God's punishment. Hence, they accepted the condition because life belongs to God and they must be thankful no matter what hssed (Wallin & Ahlström, 2010). Health was perceived as a gift from God and individuals only shared in the responsibility of maintaining the good life while God could also take it back as he is in ultimate control, both to protect individuals from getting ill and to decide who gets cured (Wallin & Ahlström, 2010). Depending on their faith background, most non‐diabetic participants stated that they would engage in prayer and believe in miracles for their healing if diagnosed (Issaka et al., 2016).

### 6.3 Consequences of poor perceptions and knowledge of diabetes

#### 6.3.1 Increased diabetes symptoms and risk of complications

The number of symptoms reported across studies ranged from 1 to 12 (Stover et al., 2001; Wallhagen & Lacson, 1999) with an average of four symptoms (Calvin et al., 2011; Stover et al., 2001). The most common symptoms were increased hunger, tinging/numbness and blurred vision. Most studies reported elevated levels of glycated haemoglobin (HbA1c), microalbuminuria and high blood pressure among diabetic participants (Calvin et al., 2011; Foster et al., 2016; Hyman et al., 2014; Mann et al., 2009; Stover et al., 2001).

Complications such as end stage renal disease (Calvin et al., 2011), neuropathy/peripheral vascular disease (PVD) (Stover et al., 2001; Wallhagen & Lacson, 1999), feet/finger and upper extremity pain (Stover et al., 2001) and amputation (Ford et al., 2002) were increased in diabetic participants with higher HbA1cs. Participants with more than one complication experienced more issues with pain (Stover et al., 2001). Perceptions of general health, and social and physical functioning were poor in the presence of neuropathy/peripheral vascular diseases (PVD) (Stover et al., 2001; Wallhagen & Lacson, 1999). Some participants expressed a sense of loss from reduced mobility resulting from amputation, decreased or loss of sexual drive (Ford et al., 2002) and vision (Ford et al., 2002; Foster et al., 2016). Diabetic retinopathy increased with the duration of diabetes.

### 6.4 Beliefs about the causes of diabetes

#### 6.4.1 Cultural and environmental effect

Culture and weather change were seen as the causative agents of diabetes by some Caribbean immigrants (Brown et al., 2007). Some argued that their lives before migration were healthy because of the living conditions in their homeland (Brown et al., 2007; Hyman et al., 2014; Issaka et al., 2016; Yeoh & Furler, 2011). They believed that their active and strenuous lifestyle in their home country (Brown et al., 2007; Issaka et al., 2016; Yeoh & Furler, 2011), coupled with the hot weather, helped to burn off excess calories (Brown et al., 2007). However, ease of transportation (Hyman et al., 2014; Issaka et al., 2016; Yeoh & Furler, 2011), unemployment (Yeoh & Furler, 2011) and long hours watching television led to social isolation in the host‐country (Brown et al., 2007; Issaka et al., 2016; Yeoh & Furler, 2011). This, they believed, brought about decreased activity that facilitated their developing diabetes. Most African participants perceived diabetes as a disease of wealthy urban people in Africa because of their luxurious easy lifestyle (Issaka et al., 2016). Some stated that they ate traditional, natural, healthy food before migration (Brown et al., 2007; Hyman et al., 2014; Issaka et al., 2016). Most noted that the scarcity of their preferred cultural food and the unfamiliarity with the available excess high carbohydrate diet in their new country compounded their dietary problem (Hyman et al., 2014; Issaka et al., 2016; Yeoh & Furler, 2011). Some Somali participants stated that they could not develop diabetes back home due to food scarcity (Issaka et al., 2016).

#### 6.4.2 Racial discrimination and stress

Most of the studies discussed participants' beliefs about the role of stress in developing diabetes and poor glycaemic control (Breland et al., 2013; Brown et al., 2007; Cooper & Lemonde, 2016; Issaka et al., 2016; Yeoh & Furler, 2011). Sources of stress included displacement from their homeland (Yeoh & Furler, 2011), migration, acculturation and adaptation to new unfamiliar environments (Brown et al., 2007; Issaka et al., 2016; Yeoh & Furler, 2011), poor living conditions and racism (Brown et al., 2007). Stress associated with racism and poverty was perceived as the main cause of poor eating habits and diabetes; for example, less nutritious foods that could not be sold in white or more affluent neighbourhoods were sent to the minority neighbourhoods (Breland et al., 2013). Other sources of stress included work (Cooper & Lemonde, 2016) and unpleasant experiences with the healthcare system (Breland et al., 2013; Brown et al., 2007; Yeoh & Furler, 2011). Such unpleasant experiences were perceived as due to race, ethnicity, language differences, their poor economic status and suboptimal healthcare (Breland et al., 2013).

#### 6.4.3 Communication and information sharing

The role of information‐sharing on glycaemic control between healthcare providers and diabetic individuals was explored (Balls‐Berry et al., 2015; Peek et al., 2010). Information was used or rejected based on its source and manner of delivery (Balls‐Berry et al., 2015; Breland et al., 2013; Brown et al., 2007; Peek et al., 2010; Yeoh & Furler, 2011). Some information was believed to be inadequate (Brown et al., 2007; Hyman et al., 2014) or racially influenced (Breland et al., 2013; Peek et al., 2010; Yeoh & Furler, 2011).

Physicians, patients and socioeconomic factors like health insurance were identified as barriers to effective communication and shared decisions between physicians and patients (Peek et al., 2010). Healthcare professionals were perceived as not providing enough information; for example, on medical conditions, not explaining test results, not paying attention to patients due to conflicting cultural views and stereotypes, dominating the conversation with little regards to patients, not reviewing treatment options with them, and making treatment decisions without considering patients' preferences. Conversely, patients were viewed by some participants as being unable to share information with their physicians especially about health concerns and medication use due to poor communication resulting from limited education, negative attitudes, beliefs and behaviours and poor participation in practices that foster good health. An erroneous belief of less self‐worth grounded in internalized racism, which makes them unable to voice their opinions and question the providers' recommended treatments, were most likely to lead to non‐compliance with the recommended treatment (Peek et al., 2010).

Some obtained information about symptoms, complications, medication and diet adjustment by observing affected individuals (Balls‐Berry et al., 2015; Skelly et al., 2006). Some relied on friends, colleagues, families and their community leaders for diabetes information (Balls‐Berry et al., 2015). Where illness conditions were not openly discussed by the affected individuals with relatives, as was noted in some studies (Balls‐Berry et al., 2015; Cameron et al., 2018; Skelly et al., 2006), awareness of the implications of the condition could be hindered as opposed to if they shared their experiences (Balls‐Berry et al., 2015).

The desire to learn more about the causes, prevention and management of the disease was expressed by some. They suggested that there was a need to consider the culture and age of people when structuring culturally targeted appropriate messaging systems that would capture the attention of the intended audience as well as the importance of healthcare providers visiting communities for better understanding of the people (Balls‐Berry et al., 2015).

## 7 DISCUSSION

Understanding perceptions and beliefs about diabetes are critical to developing effective interventions to improve diabetes‐related outcomes. To our knowledge, this is the first systematic literature review describing perceptions and beliefs about diabetes, causes and diabetes management interventions of people of African descent and the impact of these perceptions on diabetes self‐care. Our findings were consistent with that of Dimova et al. (2019) who reported that perceptions of type 2 diabetes influenced self‐management behaviours. Similarly, Mousavizadeh et al. (2018), Ledford et al. (2019) and Dimova et al. (2019) observed that diabetes perceptions underpinned by social and cultural contexts influence the uptake of recommended self‐care activities.

Studies involving other ethnic minority groups such as Kurdish immigrants (Abuelmagd et al., 2019) and migrants from Turkey, Morocco, Iraq and Curacao (Jager et al., 2019) identified similar findings. Abuelmagd et al. (2019) explored the experiences of managing type 2 diabetes, and the need for medical information of Kurdish immigrants in Norway, finding that they made changes to their diets with difficulty and neglected the need to improve their physical activity. Jager et al. (2019) explored the views of ethnic minority patients about healthy diet and diabetic care and highlighted that their ideas of healthy diets were influenced by their cultural beliefs about the benefits of particular foods. This underscores the need to understand people of African descents' dietary and exercise beliefs and practices and to base their dietary and exercise advice on this knowledge.

Varied emotions such as shock, fear and anxiety were expressed by some participants on diabetes diagnosis and diverse strategies were adopted to alleviate those emotions. For some immigrant participants this included the belief that diabetes management information and medications, which were either scarce and/or expensive in their homelands, were available and accessible in their host countries. Some used denial and ‘refusal to acquire diabetes knowledge’ to forget their diagnosis. Other studies have identified similar findings; for instance, Gonzalez et al. (2019) identified emotional burdens and coping strategies in their study exploring experiences of type 2 diabetes diagnosis and management among adults in rural Dominican Republic. As in our review, their participants found solace from the stress of their diagnosis in the fact that diabetes care and free medication services were available and by not thinking about diabetes. Quiñonez‐Tapia et al. (2019) identified that study participants went through a process involving affirmation and denial to validate their experiences, to better understand their conditions and implement behaviours to manage their health.

Our results revealed that diabetes perceptions significantly influenced beliefs and adherence to medication as was also identified by Urata et al. (2019). Insulin prescription was perceived as worse diabetes while management with diet was understood to be a lesser condition.

We identified fatalistic beliefs that emanated from illness and health beliefs and affected diabetes outcomes. Sukkarieh‐Haraty et al. (2019) revealed that diabetes fatalism and emotional distress were significantly associated with high HbA1c while diabetes education, advanced age and female gender had a positive impact on HbA1c. Similarly, San Diego et al. (2020), explored the interaction of diabetes knowledge and health fatalism as predictors of type 2 diabetes preventive behaviour. Their findings included the association of higher diabetes knowledge with healthier diet in individuals with low fatalism and the association of moderate or high diabetes knowledge with poor diet in those with greater fatalism. Other factors influencing development of type 2 diabetes were acculturation, perceived racial discrimination (Bilal et al., 2020), poverty and the associated stress (Breland et al., 2013). Racism, poverty and stress were perceived by most African Americans as leading to poor eating habits of low quality foods available in ethnic minority neighbourhoods (Breland et al., 2013). Type 2 diabetes was perceived as the disease of the rich in most studies with non‐US participants. The resultant effects of poor diabetes perceptions and beliefs were suboptimal diabetes control and increased risks of developing complications. Thus, diabetes perceptions and beliefs of people of African descent should be understood and incorporated into their diabetes discussions.

Analysis of the primary research studies included in this systematic review revealed that nine different topics concerning type 2 diabetes were covered. Most of the primary studies were descriptive/exploratory in design, with only one randomized controlled trial (Table 1), leaving a large gap in the science, impeding adequate understanding of diabetes perceptions and behaviours of Africans who live in high‐income countries. There is an urgent need, therefore, for more randomized controlled trials examining this topic with both homogenous and broad samples of African migrants to reduce bias and to close the gap in the science. The results generated by such studies must be translatable into evidence‐based clinical practice (Hariton & Locascio, 2018; Spieth et al., 2016) and have a positive impact on this populations' diabetes' management.

Programmes are needed to empower people of African descent to be more involved in their health management, to ask questions about their conditions and available treatment options when they visit their healthcare providers and to seek second opinions when they feel the need to do so. Equally, healthcare providers should be ready to address any concerns that patients from this population might have and be able to explain things in ways that they will most likely understand. Ways of improving social support for delivering accurate information about diabetes and its management are worth exploring because of the high dependency of people of African descent on families, friends and colleagues for diabetes negotiations. Such interventions may help to bridge the diabetes health inequalities that currently exist between this population and their counterparts.

## 8 LIMITATIONS

Most of the studies in this review were conducted in the USA, making African Americans the largest percentage of participants. Therefore, most of the findings from this review are from the perspective of the African American population whose sociocultural backgrounds and experiences might be different from other people of African descent. Most participants were female and we limited our search to studies with participants 18 years of age and older. Males and younger participants may perceive type 2 diabetes differently. The literature search for the included studies for this review followed a systematic and rigorous process, but we did not include grey literature, so some relevant studies may have been missed, impacting the findings. Given that eight of 26 (about 30%) of included studies were of weak to moderate quality, this may have implications for data analysis and findings. Future systematic literature review on this topic may include only articles of high quality, and/or consider only participants from African American or African Caribbean or African immigrant communities.

## 9 CONCLUSION

This review explored evidence from primary studies of diverse designs about perceptions, beliefs and management of type 2 diabetes of people of African descent living in high‐income countries. The findings demonstrate that the incidence and rate of complications of type 2 diabetes among this population could be due to poor perception and management. This has important implications for policymakers and healthcare providers, nurses in particular. Basing their diabetes interventions for people of African descent on understanding of their diabetes perceptions may be more effective with successful outcomes.

## CONFLICT OF INTEREST

There are no conflict of interest in this study.

10

### PEER REVIEW

The peer review history for this article is available at https://publons.com/publon/10.1111/jan.15266.

## Supporting information


Appendix S1
