# Supplementary material for: Systematic review: Perceptions of type 2 diabetes of people of African descent living in high‐income countries
Source: J Adv Nurs. 2022 Apr 20;78(8):2277–89. doi: 10.1111/jan.15266 (PMC9546182; doi:10.1111/jan.15266)
Supplement: Supplementary file 1 — Appendix S1 [file JAN-78-2277-s001.docx]

Appendix 1. Search strategy

| Search terms | Inclusion and exclusion criteria | Data extraction criteria |
| --- | --- | --- |
| Africans, Blacks, ethic, minority, racial, immigrant, perception, views, opinion, knowledge, understanding, beliefs, behaviours, attitude, diabetes, Diabetes Mellitus type 2, Diabetes Mellitus type 11, Non-insulin dependent diabetes (NIDD), Type 2 diabetes, Adult onset diabetes | All original research studies published in English language that included people of African descent over 18 years of age, regardless of whether they had diabetes or not were included.  Systematic, scoping, and literature reviews, commentaries, opinions, editorials, and studies focused on diabetes other than type 2 were excluded. | Author, year, aim, method, ethnicity, country of study, summary of findings and limitations |
